# The Postmortem Interval of Two Decedents and Two Dog Carcasses at the Same Scene Based on Forensic Entomology

**DOI:** 10.3390/insects13020215

**Published:** 2022-02-21

**Authors:** Liangliang Li, Yinghui Wang, Mingqing Liao, Yanan Zhang, Chengtao Kang, Gengwang Hu, Yi Guo, Jiangfeng Wang

**Affiliations:** 1 Department of Forensic Medicine, Soochow University, Suzhou 215000, China; 20214021012@stu.suda.edu.cn (L.L.); 20194221064@stu.suda.edu.cn (Y.W.); 20204221029@stu.suda.edu.cn (Y.Z.); 20214221013@stu.suda.edu.cn (C.K.); 20214221010@stu.suda.edu.cn (G.H.); 20214221021@stu.suda.edu.cn (Y.G.); 2 Key Laboratory of Evidence Identification in Universities of Shandong Province, Shandong University of Political Science and Law, Jinan 250014, China; 3 Criminal Police Branch, Zhongshan Public Security Bureau, Zhongshan 528400, China; zsfylmq@163.com

**Keywords:** forensic entomology, minimum PMI, multiple human corpses, dog carcasses

## Abstract

**Simple Summary:**

This paper reports a case in which the minimum postmortem interval (PMI) of two corpses, a man and a woman, and two dog carcasses at the same scene was estimated using forensic entomology. The corpses were found in various states of decay and had been colonized by different insect species. A total of eight taxa of immature insects were isolated from the four corpses and carcasses. The minimum PMIs were estimated to be about 8.75 days for the corpse of the woman, 4.17 days for that of the man, 3.13 days for the dog carcass found in the stairwell, and about 28.80 days for the dog carcass found in the toilet. These estimations were consistent with other evidence. Although the soft tissue loss observed on the man’s corpse was more severe than that of the woman’s corpse, the woman had died much earlier than the man. The discrepancy is thought to have been caused by dogs’ feeding activity. This case report provides a reference point and new perspectives for forensic entomology research on estimating the minimum PMIs of multiple human corpses and animal carcasses found in an indoor environment.

**Abstract:**

In this paper, we report the estimation of the minimum PMIs of two human corpses and two dog carcasses using entomological evidence. Corpses of an elderly couple and carcasses of four dogs were found scattered on different floors in a house. The scene was very dirty. In addition, there were 12 emaciated live dogs at the scene. The corpses had been eaten by the dogs to different degrees, but the damage was greater on the man’s corpse. After forensic examination, it was concluded that both individuals died of natural causes. The minimum PMIs of the two individuals and the two dogs were estimated using entomological evidence. The minimum PMIs of the other two dogs were not estimated because of the risk of contamination with the human corpses. Different insect species were found on each of the corpses and carcasses. The minimum PMIs were estimated as about 8.75 days for the woman, 4.17 days for the man, 3.13 days for the dog found in the stairwell and about 28.80 days for the dog found in the toilet. These estimations coincided with the time the woman stopped communicating with her daughter and when the electricity consumption at the house decreased significantly.

## 1. Introduction

Medicocriminal entomology, one of the three principal areas of forensic entomology, mainly utilizes the growth and development stage as well as the succession of significant insects and arthropods to estimate the postmortem interval (PMI) [1]. Based on the development data of Calliphoridae, Sarcophagidae, Muscidae, Stratiomyidae, and certain beetles isolated from corpses, the minimum PMI can be calculated [2]. Many factors, including temperature, relative air humidity, and exposure to the sun and rain, affect the development of necrophagous insects and the decay rate of corpses [3].

Cases involving forensic entomology are complex and diverse. Forensic scenes may be indoor, outdoor, in water, etc., and the condition of corpses also differs and may include being exposed, wrapped, buried, intact, or dismembered [4,5]. Different scenes and corpse conditions have variable effects on insect evidence, and each case is unique. Hence, it is considered important to report each case because it can provide a reference point for future investigations of similar cases. To date, numerous classic cases of successful application of forensic entomology have been reported worldwide [6,7,8,9,10]. These cases not only provide references for the application of entomology but also provide insights for further research. 

Forensic entomology can not only be used to estimate the minimum PMI of human corpses but also the minimum PMI of wild animals and pets [11,12,13,14]. Since most of the forensic entomological succession and insect development experiments are carried out on animal carcasses or tissues, the methods developed in such research can readily be used to estimate the minimum PMI of animals. In addition, comparative experiments have illustrated that the insect fauna of human and animal corpses does not differ considerably. Also, the composition of arthropod species on corpses or carcass is similar, and the stages of colonization follow the same pattern [15,16]. Thus, entomological research methods may be applied in veterinary medicine to determine the time of death of animals [15]. Dogs are the pets most commonly kept by human beings. With the development of society, there are more and more people living alone, especially the elderly population. As such, most of them keep dogs as companions. If the death of a pet owner goes unnoticed, the domestic pets are left unattended. Because of hunger, the pets may feed on the corpses of their owners and may eventually die if not saved on time [17]. In such cases, the influence of pets on the decomposition of the corpses should be considered, and sometimes, the time of death of pets should also be estimated [18]. Case reports in the above special conditions are extremely important and their use can promote forensic entomology constantly evolving and growing.

This paper presents the case of two human corpses and carcasses of four dogs. The corpses and carcasses were found in different states of decomposition and colonized by different insect species. In addition, the activities of 12 surviving dogs at the scene were apparent and significantly impacted the state of the corpses or carcasses and the environment. We estimated the minimum PMIs of the corpses of the two people and carcasses of the two of the four dogs. This case not only focuses on an estimation of the time of death but also demonstrates the presence of specific insects on the corpses or carcasses under certain circumstances.

## 2. Case Report

### 2.1. Case Description

**Figure 1:**
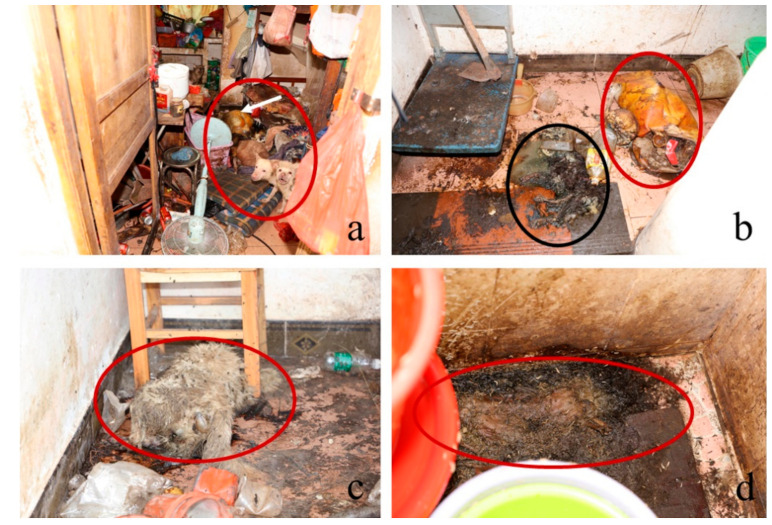
Overview of forensic scene: (**a**) Woman corpse; (**b**) Man corpse (red circle) and the carcass of one of the dogs (black circle); (**c**) Carcass of a dog on the first floor; (**d**) Carcass of a dog in the toilet on the second floor.

**Figure 2:**
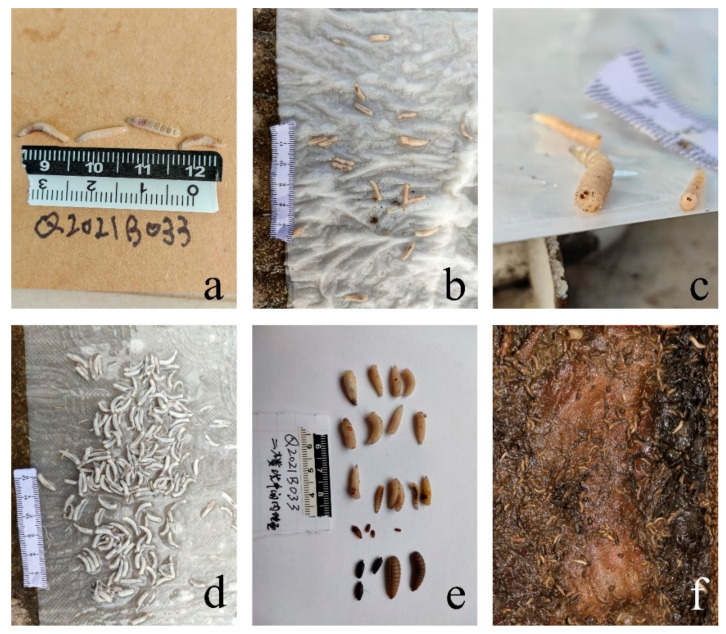
Insects found on corpses and dog carcasses: (**a**) on the woman corpse; (**b**,**c**) on the man corpse; (**d**) on the dog carcass on the first floor; (**e**,**f**) on the dog carcass in the toilet on the second floor.

On 22 July 2021, two human corpses and four dog carcasses were found inside a three-story residential house in Zhongshan City (22°30′59.33″ N, 113°23′33.92″ E), Guangdong province. The windows of the house were partially closed. The corpse of the woman was on the floor beside the bed in the bedroom on the second floor, laying in a supine position (Figure 1a), and was in a state of high decomposition with a skeletonized head and swollen abdomen. The feet were missing, and based on the bite mark, were thought to have been eaten by dogs. The corpse of the man was found on the ground, at the turn of a set of stairs between the first and second floors (Figure 1b). The corpse had also been partially eaten by dogs, but the head had completely detached. Only the bones remained of the upper limbs, and the tissues of the buttocks and legs were missing. Neither corpse was dressed. The carcass of one of the dogs was found near the man’s corpse (the black circle in Figure 1b, not evaluated for PMI); two other carcasses were found in the living room on the first floor (Figure 1c). Since the carcass of the other dead dog was not estimated, it is not shown or discussed in this report. The fourth carcass was found in the toilet on the second floor and had completely decomposed (Figure 1d). Besides the four dead dogs, 12 live but emaciated dogs were found at the scene. The rice in the house had been eaten by the dogs. The toilet on the second floor was leaking, and a plastic bucket used to collect the leaking water was full and could be accessed by the dogs. The house was very dirty and stuffy, with household waste and dog feces scattered all over. The man was 73 years old, whereas the woman was 67 years old. A police investigation revealed that both individuals had died from natural causes, which ruled out the possibility of homicide or suicide. The police collected insects from both the corpses (Figure 1a,b) and carcasses (Figure 1c,d), which were then identified at the Laboratory of Forensic Entomology of Soochow University. The developmental stage of the insects was also estimated (Figure 2 and Table 1).

### 2.2. Meteorological Data

**Figure 3:**
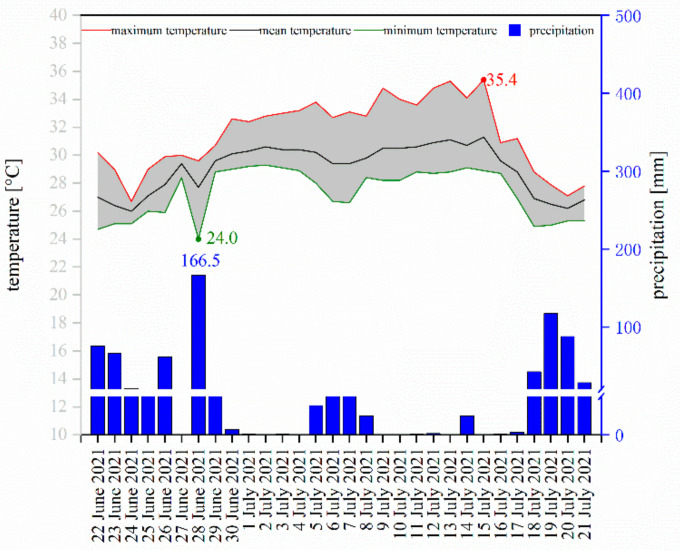
Daily temperature and precipitation change from 22 June 2021 to 21 July 2021.

According to a nearby weather station, the total average temperature ten days before the corpses were discovered was 28.9 °C. It rained every day. The highest daily rainfall was 117.2 mm, and the average daily rainfall was 27.8 mm. The changes in temperature and precipitation 30 days before the discovery are shown in Figure 3. Within this period, the lowest temperature was 24.0 °C, whereas the highest temperature was 35.4 °C. The average temperature over 30 days was 29.1 °C. The indoor air conditioner was off. 

### 2.3. Insect Evidence and PMI Estimation

Insect samples were collected from the scene according to the method described by Amendt et al. [22] and were examined immediately in the laboratory (within 2 h). The woman’s corpse had been colonized by three insect species, whereas only two insect species were isolated from the man’s corpse. Only one insect species had colonized the dog carcass in the stairwell, whereas six insect species were identified and one of Sarcophagidae not identified had colonized the dog carcass in the toilet. Briefly, the larvae were placed in hot water >80 °C for 30 s and then stored in 75% ethanol. Larger individuals were selected by visual judgment. The body length of the larvae was measured using a digital vernier caliper with an accuracy of 0.01 mm (Shengong, Shanghai). The 8 taxa of immature insects collected from the corpses and carcasses were identified according to Fan’s identification key [23], other recently published keys [24,25], and references [19,26,27,28,29]. All the insects belonged to the order Diptera (Table 1). The specific insects on each corpse and carcass are shown in Table 1 and Figure 2. The method and data of every insect species to estimate the minimum PMI of two human corpses and two dog carcasses can be seen in Supplementary Figure S1.

The minimum PMI was estimated using the oldest insects. The maximum length of *Chrysomya nigripes* Aubertin (Diptera: Calliphoridae) larvae isolated from the woman’s corpse was 11.68 mm and according to unpublished data from our laboratory, its development time is about 210 h (8.75 days). Considering that the development time of *C. nigripes* was the longest, it was used to estimate the minimum PMI. Accordingly, the minimum PMI of the woman’s corpse was about 8.75 days. 

The *Chrysomya megacephala* (F.) (Diptera: Calliphoridae) isolated from the man corpse was shortened and about to pupate. At 28.9 °C, according to the development data by Wang [19], the time required by the insect to develop from egg to the shorten pre-pupation stage is 100 h. When used as the indicator insect, the minimum PMI of the man corpse was estimated not to be less than 100 h (4.17 days). 

The larvae of *C. megacephala* were found on the dog’s carcass on the first floor. They were 15.26 mm long and according to the development data by Wang [19], a developmental time of 75 h (3.13 days), the minimum PMI of the dog was estimated not to be less than 75 h (3.13 days). Many insect species were isolated from the dead dog in the toilet on the second floor. According to the development data by Wang [19], it takes about 691 h (28.80 days) for the *Hermetia illucens* (L.) (Diptera: Stratyiomidae) larvae to develop from an egg to pre-pupae. Thus, *H. illucens* was used as the indicator insect for the minimum PMI of the dog, which was estimated to be about 28.80 days.

### 2.4. History of the Couple 

Both the man and the woman suffered from various senile diseases and cognitive impairment. The man was paralyzed and, thus, mostly bedridden. He depended mainly on the woman. The couple had adopted 16 local Chinese rural dogs (Tugou). The daughter of the woman had sent her money through the mobile phone on July 12th. Thereafter, there was no contact between the woman and her daughter or other people outside the house till the corpses were discovered, which coincided with our estimated minimum PMI. In addition, the electricity consumption on the smart meter at the premise was generally 2–3 units per day but decreased to only 0.26 units/day after July 12th (Table 2). This indicates that the household appliances were not used after this date, consistent with our estimation of the time of death. The mobile phone of the man was found under the bed of the woman. The police speculated that the man could not find the mobile phone to call for help after the death of the woman, and he subsequently died of illness, neglect, and starvation. The samples in 75% ethanol are kept at the evidence Appraisal Room of the Criminal Police Branch, Zhongshan Public Security Bureau, and the 12 surviving dogs were euthanized by the police. 

## 3. Discussion

Herein, we report the use of forensic entomology to estimate the minimum PMI. The home of the deceased persons had no surveillance cameras. The case raised the question of both the police and family members, given the mystery surrounding the death of the couple. After ruling out any criminality, accurate estimation of PMI became important in revealing events in the buildup to the deaths of the couple. According to the estimated minimum PMI and other physical evidence, we can make the following assumptions about this incident: the woman fell ill and died, and the man, being disabled, couldn’t find a phone to call for help. After four days, the man also fell ill and died because he was left unattended. After the man and the woman died, the fifteen dogs trapped in the house initially ate the rice since their owners had previously fed them rice. The dogs also drank water from the toilet. After finishing the rice, the dogs began to feed on the corpses of the man and woman. In this case, two human corpses and four dog carcasses were found at the same scene. The dog in the toilet had entered an advanced stage of decay, and its minimum PMI indicated that it died while the owners were alive. This dog likely died of illness, and the carcass was not disposed of because the owner was cognitively impaired. The other three dead dogs, although very thin, did not starve to death but were seriously injured and died in the process of competing with other dogs for food. The man had been eaten by the dogs more severely than the woman. Analysis showed that the dogs fed on the corpse of the man first, and the bloody smell caused by subsequent physical injuries attracted the dogs to feed on the man’s corpse mainly. To our surprise, the living dogs did not feed on the carcasses of the dead dogs [30].

Although located in the same building, different insect compositions appeared on different corpses. The larvae of *C. megacephala*, *Musca domestica* L. (Diptera: Muscidae), and *C. nigripes* were found on the corpse of the woman, and the larvae of *C. megacephala* and *M. domestica* were found on the corpse of the man. Only the larvae of *C. megacephala* were found on the carcass of the dog on the first floor. Most insect species were discovered on the dog carcasses on the second floor, including the immature stage of *C. megacephala*, *C. nigripes*, *Chrysomya rufifacies* (Macquart) (Diptera: Calliphoridae), *Hydrotaea spinigera* (Stein) (Diptera: Muscidae), *Megaselia spiracularis* Schmitz (Diptera: Phoridae), *H. illucens* and an unidentified Sarcophagidae species. The longer the estimation of minimum PMI, the greater the variety of insects, with six species of insects appearing on the remains of the dog on the second floor. This may be related to the different PMIs and the locations of the corpses in the building. Different insects colonize corpses at different time points. During the period after death, insects would gradually be attracted by the smell, and the number and species of insects on the corpse would subsequently increase [31]. No beetles were found on the corpses at the scene, which might be related to the heavy rainfall during the time of the incident. In addition, the insect species identified on the corpses of the humans and the dog found on the first floor of the building were fewer; the reason for this may be that the surviving dogs were constantly moving around the corpses, thereby affecting the colonization of insects. Not only will the constant activities of dogs affect the insect oviposition activity, but the dogs may also eat the corpse tissues infested with maggots.

The insects found at the scene of this case are species commonly found on corpses, and all of these species were reported in the succession experiments undertaken in the area where the incident happened [15]. According to data from our many years of field succession research [15], in the summer in this region, *C. megacephala* and *C. rufifacies* are the first wave insects to reach a corpse, while *C. nigripes* and *Hy. spinigera* colonize corpses and lay eggs on the 2nd-3rd day, which is consistent with Malaysian research reports [32]. In this case, the colonization time of *C. megacephala* and *C. rufifacies* was later than that of *C. nigripes*, and the reason for this needs further investigation. Compared with the Calliphoridae, Phoridae is relatively small and can therefore easily enter a room [33]. Adults of Phoridae prefer a humid environment, such as rotten plants, animal corpses, flowers, fungi, etc., and their larvae feed on decaying substances, such as animal carcasses, decaying plant material, and animal excrements [24]. In addition, they are one of the most common flies found on corpses and animal carcasses indoors [34]. In the present case, this was proved by the appearance of *Me. spiracularis*. *Musca domestica* is a species ecologically associated with human beings, which mainly appears on livestock farms, dung yards, household waste stations, etc., and is occasionally found during the entomological examination of indoor cases. Many case examinations and succession studies have shown that the adult housefly will appear on corpses, but its reproduction on carcasses has rarely been reported. Also, it is usually isolated from indoor corpses in dirty conditions [20,35,36]. In the present case, the *M. domestica* larvae were found on both the man and woman corpses, and the main reasons were the poor sanitary conditions at the scene, with a large amount of household waste and dog feces, which encourages *M. domestica* to reproduce and colonize corpses. According to our research, *H. illucens* is often the last wave of insects to reach a corpse [15]. In the present case, the larvae of *H. illucens* were found only on the carcass of the dog found in the toilet, suggesting that this carcass had the longest minimum PMI.

Even for the owners who feed and take care of their pets, pets will feed on the corpses of their owners after death in response to extreme hunger, as has been reported previously [17]. Similar to our case, three of the cases reported by Sanford [18] involved an indoor forensic scene of dead humans and animals. The minimum PMIs of both the corpses and carcasses were estimated using insect evidence. However, the cases reported by Sanford differ from our case in a way. For instance, our case is more complex, involving two human corpses and two animal carcasses. Contrary to our case, the number of species on human corpses was more than on the carcasses. The reason for this difference may be that in all the three cases reported at Sanford [18], the owners’ minimum PMI was earlier than the pets, whereas in our case, it is presumed that the minimum PMI of the dog in the toilet may have been earlier than that of the man and woman corpses. Therefore, the earlier the minimum PMI of the corpse, the more insect species that are attracted. 

In the present case, the minimum PMIs of two human corpses and two dog carcasses were successfully estimated according to the entomological evidence. The minimum PMIs were calculated according to the meteorological temperature, which was a limitation to the estimation of the minimum PMI in this case. Considering that maggots near corpses may crawl to feed on other corpses and complicate the minimum PMI estimation, we did not calculate the minimum PMI of dogs near the man corpse. To take this factor into account when calculating this minimum PMI, we would first use DNA-based analysis of the stomach contents of the maggots to determine their original host before carrying out the calculation [37]. In addition, although maggots on the man’s corpse may have come from the dog carcasses, the man died earlier than the dogs, and we calculated the minimum PMI based on the oldest immature insects, so there was no influence on the estimation of the minimum PMI. At the same time, this case also suggests that research on forensic entomology should focus on minimum PMI estimates for corpses located indoors. In subsequent research, we should pay attention to two key points. Firstly, we should determine whether there is cross-contamination between insect evidence on different corpses in the same room. Secondly, since many of the corpses at the same scene died successively, we should determine whether the insect succession wave on the corpse that died first will be influenced by the insects present on the corpses where the death occurred later.

## 4. Conclusions

In the case described, two human corpses and two animal carcasses were found at the same scene, and the composition of insects associated with the remains was different. Dogs had mutilated the corpses and interfered with the scene. We used the insect with the longest development time on each corpse and carcass to estimate minimum PMI. Thus, the estimation of the minimum PMI for several corpses and carcasses also differed. But these estimations were convincing based on the time when communication between the woman and her daughter stopped and when the electricity consumption at the house decreased considerably. 

## Figures and Tables

**Table 1:** The insects isolated on the corpses of the couple and two dog carcasses. The minimum PMIs were estimated as about 8.75 days for the woman, 4.17 days for the man, 3.13 days for the dog found in the stairwell and about 28.80 days for the dog found in the toilet.

Corpses or Carcasses	Family	Insect Species	Development Stage (Number of the Samples)	Indicator	Minimum PMI
Corpse of the woman	Calliphoridae	*Chrysomya megacephala*	Larvae, 3rd instar (15)	Wandering larvae with shorten body which is about to pupation	100 h (4.17 d) [19]
		*Chrysomya nigripes*	Larvae, 3rd instar (11)	The maximum larval body length is 11.68 mm	210 h (8.75 d) [*]
	Muscidae	*Musca domestica*	Larvae, 2nd instar (3), 3rd instar (10)	The maximum larval body length is 11.22 mm	70 h (2.92 d) [20]
Corpse of the man	Calliphoridae	*Chrysomya megacephala*	Larvae, 3rd instar (16)	Wandering larvae with shorten body which is about to pupation	100 h (4.17 d) [19]
	Muscidae	*Musca domestica*	Larvae, 2nd instar (2), 3rd instar (9)	The maximum larval body length 10.91 mm	70 h (2.92 d) [20]
Dog carcass in the stairwell	Calliphoridae	*Chrysomya megacephala*	Larvae, 3rd instar (20)	The maximum larval body length is 15.26 mm	75 h (3.13 d) [19]
Dog carcass in the toilet	Calliphoridae	*Chrysomya megacephala*	Larvae, 3rd instar (11)	The maximum larval body length is 14.10 mm	71 h (2.96 d) [19]
		*Chrysomya nigripes*	Larvae, 3rd instar (10; Puparia (3)	Dark puparia	/
		*Chrysomya rufifacies*	Larvae, 3rd instar (10)	The maximum larval body length is 12.79 mm	107 h (4.46 d) [19]
	Muscidae	*Hydrotaea spinigera*	Larvae, 2nd instar (3), 3rd instar (13)	The maximum larval body length is 12.07 mm	90 h (3.75 d) [19]
	Phoridae	*Megaselia spiracularis*	Pupae (8); Empty puparia (5)	Empty puparia	273 h (11.38 d) [21]
	Sarcophagidae	NI	Larvae, 3rd instar (3)	The maximum larval body length is 17.31 mm	/
	Stratiomyidae	*Hermetia illucens*	Larvae, 3rd instar (6); Pre-pupae (3)	Pre-pupae	691 h (28.80 d) [19]

“*” represents unpublished data (Supplementary Figure S1), and “NI, species not identified” and “/, minimum PMI not determined”. Minimum PMI estimated based on literature and data shown in the Supplementary Figure S1.

**Table 2:** Electricity consumption data on the smart meter at the dead couple’s home.

Date	Electric Meter Initial Value	Electric Meter End Value	Daily Electricity Consumption
14 June 2021	5542.26	5545.46	3.2
15 June 2021	5545.46	5547.77	2.31
16 June 2021	5547.77	5550.86	3.09
17 June 2021	5550.86	5553.78	2.92
18 June 2021	5553.78	5556.67	2.89
19 June 2021	5556.67	5560.17	3.5
20 June 2021	5560.17	5563.25	3.08
21 June 2021	5563.25	5565.27	2.02
22 June 2021	5565.27	5567.8	2.53
23 June 2021	5567.8	5569.68	1.88
24 June 2021	5569.68	5572.1	2.42
25 June 2021	5572.1	5575.9	3.8
26 June 2021	5575.9	5578.32	2.42
27 June 2021	5578.32	5580.47	2.15
28 June 2021	5580.47	5583.61	3.14
29 June 2021	5583.61	5585.68	2.07
30 June 2021	5585.68	5587.99	2.31
1 July 2021	5587.99	5589.8	1.81
2 July 2021	5589.8	5592.18	2.38
3 July 2021	5592.18	5595.23	3.05
4 July 2021	5595.23	5597.88	2.65
5 July 2021	5597.88	5601.16	3.28
6 July 2021	5601.16	5603.78	2.62
7 July 2021	5603.78	5606.39	2.61
8 July 2021	5606.39	5608.1	1.71
9 July 2021	5608.1	5610.37	2.27
10 July 2021	5610.37	5612.7	2.33
11 July 2021	5612.7	5614.18	1.48
12 July 2021	5614.18	5614.44	0.26
13 July 2021	5614.44	5614.71	0.27
14 July 2021	5614.71	5614.97	0.26
15 July 2021	5614.97	5615.23	0.26
16 July 2021	5615.23	5615.5	0.27
17 July 2021	5615.5	5615.77	0.27
18 July 2021	5615.77	5616.03	0.26
19 July 2021	5616.03	5616.3	0.27
20 July 2021	5616.3	5616.58	0.28

## Data Availability

Not applicable.

## Supplementary Materials

The following supporting information can be downloaded at: https://www.mdpi.com/article/10.3390/insects13020215/s1. File S1: The method and data of every insect species used to estimate the minimum PMI of two human corpses and two dog carcasses. References [38,39,40,41] are cited in the supplementary materials.

## References

[1] Huntington T.E., Weidner L.M., Hall R.D., Byrd J.H., Tomberlin J.K. (2019). Introduction: Current perceptions and status of forensic entomology. Forensic Entomology: The Utility of Arthropods in Legal Investigations.

[2] Amendt J., Goff M.L., Campobasso C.P., Grassberger M. (2010). Current Concepts in Forensic Entomology.

[3] Campobasso C.P., Di Vella G., Introna F. (2001). Factors affecting decomposition and Diptera colonization. Forensic Sci. Int..

[4] Kotzé Z., Aimar S., Amendt J., Anderson G.S., Bourguignon L., Hall M.J.R., Tomberlin J.K. (2021). The forensic entomol ogy case report—A global perspective. Insects.

[5] Wang Y., Wang Y., Wang M., Xu W., Zhang Y., Wang J. (2021). Forensic entomology in China and its challenges. Insects.

[6] Goff M.L., Omori A.I., Gunatilake K. (1988). Estimation of postmortem interval by arthropod succession. Three case studies from the Hawaiian Islands. Am. J. Forensic Med. Pathol..

[7] Benecke M., Josephi E., Zweihoff R. (2004). Neglect of the elderly: Forensic entomology cases and considerations. Forensic Sci. Int..

[8] Sanford M.R. (2017). Insects and associated arthropods analyzed during medicolegal death investigations in Harris County, Texas, USA: January 2013–April 2016. PLoS ONE.

[9] Corrêa R.C., Caneparo M.F.C., Vairo K.P., de Lara A.G., Moura M.O. (2019). What have we learned from the dead? A compilation of three years of cooperation between entomologists and crime scene investigators in Southern Brazil. Rev. Bras. Entomol..

[10] VanLaerhoven S.L., Merritt R.W. (2019). 50 years later, insect evidence overturns Canada’s most notorious case—Regina v. Steven Truscott. Forensic Sci. Int..

[11] Moore M.K., Frazier K. (2019). Humans are animals, too: Critical commonalities and differences between human and wild life forensic genetics. J. Forensic Sci..

[12] Brundage A., Byrd J.H. (2016). Forensic entomology in animal cruelty cases. Vet. Pathol..

[13] Listos P., Gryzińska M., Batkowska J., Dylewska M., Czepiel-Mil K. (2018). Application of research in the field of forensic entomology for determining the time of death in dogs. Med. Weter..

[14] Arnaldos M.I., García M.D., Romera E., Presa J.J., Luna A. (2005). Estimation of postmortem interval in real cases based on experimentally obtained entomological evidence. Forensic Sci. Int..

[15] Wang Y., Ma M., Jiang X., Wang J., Li L., Yin X., Wang M., Lai Y., Tao L. (2017). Insect succession on remains of human and animals in Shenzhen, China. Forensic Sci. Int..

[16] Matuszewski S., Hall M.J.R., Moreau G., Schoenly K.G., Tarone A.M., Villet M.H. (2020). Pigs vs people: The use of pigs as analogues for humans in forensic entomology and taphonomy research. Int. J. Leg. Med..

[17] Buschmann C., Solarino B., Püschel K., Czubaiko F., Heinze S., Tsokos M. (2011). Post-mortem decapitation by domestic dogs: Three case reports and review of the literature. Forensic Sci. Med. Pat..

[18] Sanford M.R. (2015). Forensic entomology of decomposing humans and their decomposing pets. Forensic Sci. Int..

[19] Wang J. (2019). Practical Forensic Entomology.

[20] Wang Y., Yang L., Zhang Y., Tao L., Wang J. (2018). Development of *Musca domestica* at constant temperatures and the first case report of its application for estimating the minimum postmortem interval. Forensic Sci. Int..

[21] Wang Y., Zhang Y., Hu G., Wang M., Zhu R., Zhai Y., Sun J., Li X., Wang L., Wu M. (2020). Development of *Megaselia spiracularis* (Diptera: Phoridae) at different constant temperatures. J. Therm. Biol..

[22] Amendt J., Campobasso C.P., Gaudry E., Reiter C., LeBlanc H.N., JR Hall M. (2007). Best practice in forensic entomology—Standards and guidelines. Int. J. Leg. Med..

[23] Fan Z. (1992). The Keys of Common Flies of China.

[24] Xue W., Zhao J. (1996). Flies of China.

[25] Liu G. (2001). Taxonomy of Phoidae in China.

[26] Sukontason K., Sukontason K.L., Ngern-Klun R., Sripakdee D., Piangjai S. (2004). Differentiation of the third instar of forensically important fly species in Thailand. Ann. Entomol. Soc. Am..

[27] Sukontason K., Piangjai S., Siriwattanarungsee S., Sukontason K.L. (2008). Morphology and developmental rate of blow flies *Chrysomya megacephala* and *Chrysomya rufifacies* in Thailand: Application in forensic entomology. Parasitol. Res..

[28] Feng D., Liu G. (2012). Morphology of immature stages of *Megaselia spiracularis* Schmitz (Diptera: Phoridae). Microsc. Res. Tech..

[29] Barros L.M., Gutjahr A.L.N., Ferreira Keppler R.L., Martins R.T. (2019). Morphological description of the immature stages of *Hermetia illucens* (Linnaeus, 1758) (Diptera: Stratiomyidae). Microsc. Res. Tech..

[30] Gonzálvez M., Martínez-Carrasco C., Sánchez-Zapata J.A., Moleón M. (2021). Smart carn ivores think twice: Red fox delays scavenging on conspecific carcasses to reduce parasite risk. Appl. Anim. Behav. Sci..

[31] Catts E.P., Goff M.L. (1992). Forensic entomology in criminal investigations. Annu. Rev. Entomol..

[32] Syamsa R.A., Omar B., Ahmad F.M.S., Hidayatulfathi O., Shahrom A.W. (2017). Comparative fly species composition on indoor and outdoor forensic cases in Malaysia. J. Forensic Leg. Med..

[33] Disney R.H. (2008). Natural history of the scuttle fly, *Megaselia scalaris*. Annu. Rev. Entomol..

[34] Zuha R.M., Ankasha S.J., Disney R.H.L., Omar B. (2016). Indoor decomposition study in Malaysia with special reference to the scuttle flies (Diptera: Phoridae). Egypt. J. Forensic Sci..

[35] Al-Qahtni A.H., Al-Khalifa M.S., Mashaly A.M. (2020). Two human cases associated with forensic insects in Riyadh, Saudi Arabia. Saudi J. Biol. Sci..

[36] Bonacci T., Vercillo V., Benecke M. (2017). Flies and ants: A forensic entomological neglect case of an elderly man in Calabria, Southern Italy. Rom. J. Leg. Med..

[37] Mohammad Z., Alajmi R., Alkuriji M., Metwally D., Kaakeh W., Almeaiweed N. (2020). Role of *Chrysomya albiceps* (Diptera: Calliphoridae) and *Musca domestica* (Diptera: Muscidae) Maggot Crop Contents in Identifying Unknown Cadavers. J. Med. Entomol..

[38] Zhang Y., Wang Y., Yang L., Tao L., Wang J. (2018). Development of *Chrysomya megacephala* at constant temperatures within its colony range in Yangtze River Delta region of China. Forensic Sci. Res..

[39] Hu G., Wang Y., Sun Y., Zhang Y., Wang M., Wang J. (2019). Development of *Chrysomya rufifacies* (Diptera: Calliphoridae) at Constant Temperatures Within its Colony Range in Yangtze River Delta Region of China. J. Med. Entomol..

[40] Wang Y., Zhang Y., Wang M., Hu G., Fu Y., Zhi R., Wang J. (2020). Development of *Hydrotaea spinigera* (Diptera: Muscidae) at Constant Temperatures and Its Significance for Estimating Postmortem Interval. J. Med. Entomol..

[41] Li L., Wang Y., Wang J. (2016). Intra-puparial development and age estimation of forensically important *Hermetia illucens* (L.). J. Asia-Pac. Entomol..

