# Supplementary material for: The Postmortem Interval of Two Decedents and Two Dog Carcasses at the Same Scene Based on Forensic Entomology
Source: Insects. 2022 Feb 21;13(2):215. doi: 10.3390/insects13020215 (PMC8876788; doi:10.3390/insects13020215)
Supplement: Supplementary file 1 [file insects-13-00215-s001.zip › insects-1564078-supplementary.pdf]

Supplementary File S1:

The method and data of every insect species used to estimate the minimum PMI of two human corpses and two dog carcasses

Corpse of the woman

Species: *Chrysomya megacephala* (F.) (Diptera: Calliphoridae)
Reference: [1,2] (The data of two references are same, only [2] was cited in text for reducing our self-citations)
Indicator: Wandering larvae with shorten body which is about to pupation
Temperature: 28.9°C
Minimum PMI: not less than 100 h

Based on isomorphen diagram of developmental stages of *Chrysomya megacephala* by Zhang et al. 2018 [1] and Wang 2019 [2], the duration from oviposition to pupation is estimated about 110 h. Samples were brought back to the laboratory for feeding and pupated about 10 h later. The minimum PMI: 110 h − 10 h = 100 h.

Species: *Chrysomya nigripes* Aubertin (Diptera: Calliphoridae)
Reference: unpublished data
Indicator: The maximum larval body length 11.68 mm
Temperature: 28.9°C
Minimum PMI: about 210 h
There is no data of 28.9°C, so only refer to data of 28°C. According to the unpublished data of our laboratory, the egg stage of *Chrysomya nigripes* is 18 h ± 0.7 h, and the development data of larvae are shown in the Table S1 below. It can be seen that the maximum body length of *Chrysomya nigripes* on the 8th day (192 h) is 11.85, which is close to our current maximum length of 11.68.
Total: 18 h + 192 h = 210 h

Table S1. Average larval body length (±standard deviation) of *Chrysomya nigriceps* at 28 °C.

Time after hatching (d/h)	Average larval body length
0.5/12	1.66 ± 0.15
1.0/24	2.14 ± 0.32
1.5/36	2.28 ± 0. 21
2.0/48	2.33 ± 0.37
2.5/60	2.43 ± 0.34
3.0/72	2.80 ± 0.29
3.5/84	3.27 ± 0.45
4.0/96	4.33 ± 0.43
4.5/108	4.84 ± 0.51
5.0/120	5.80 ± 0.48
5.5/132	6.88 ± 0.62
6.0/144	8.14 ± 0.84
6.5/156	9.57 ± 0.88

7.0/168	10.17 ± 0.71
7.5/180	11.23 ± 0.66
8.0/192	11.85 ± 0.58
8.5/204	10.93 ± 0.74

Species: *Musca domestica* L. (Diptera: Muscidae)
Reference: [3]
Indicator: Maximum larval body length 11.22 mm
Temperature: 28.9°C
Minimum PMI: not less than 70 h
Based on isomorphen diagrams by Wang et al. 2018 [3], the egg stage is about 14 h. Based on isomegalen diagram by Wang et al. 2018 [3], duration of development to length 11.22 mm is about 56 h.
Total: 14 h+ 56 h = 70 h

Corpse of the man

Species: *Chrysomya megacephala* (F.) (Diptera: Calliphoridae)
Reference:[1,2]
Indicator: Wandering larvae with shorten body which is about to pupation
Temperature: 28.9°C
Minimum PMI: not less than 100 h

Based on isomorphen diagram of developmental stages of *Chrysomya megacephala* by Zhang et al. 2018 [1] and Wang 2019 [2], the duration from oviposition to pupation is estimated about 110 h. Samples were brought back to the laboratory for feeding and pupated about 10 h later. The minimum PMI: 110 h − 10 h = 100 h.

Species: *Musca domestica* L. (Diptera: Muscidae)
Reference: [3]
Indicator: Maximum larval body length 10.91 mm
Temperature: 28.9°C
Minimum PMI: not less than 70 h
Based on isomorphen diagrams by Wang et al. 2018 [3], the egg stage is about 14 h. Based on isomegalen diagram by Wang et al. 2018 [3], duration of development to length 10.91 mm is about 56 h.
Total: 14 h + 56 h = 70 h

Dog carcass in the stairwell

Species: *Chrysomya megacephala* (F.) (Diptera: Calliphoridae)
Reference:[1,2]
Indicator: Maximum larval body length 15.26 mm
Temperature: 28.9°C

Minimum PMI: not less than 75 h

"Based on isomorphen diagrams by Zhang et al. 2018 [1] and Wang 2019 [2], the egg stage is about 11 h. Based on isomegalen diagram by Zhang et al. 2018 [1] and Wang 2019 [2], duration of development to length 15.26 mm is about 64 h.

Total: 11 h + 64 h = 75 (h)

Dog carcass in the toilet

Species: *Chrysomya megacephala* (F.) (Diptera: Calliphoridae)

Reference:[1,2]

Indicator: Maximum larval body length 14.10 mm

Temperature: 28.9°C

Minimum PMI: not less than 71 h

"Based on isomorphen diagrams by Zhang et al. 2018 [1] and Wang 2019 [2], the egg stage is about 11 h. Based on isomegalen diagram by Zhang et al. 2018 [1] and Wang 2019 [2], duration of development to length 14.10 mm is about 60 h.

Total: 11 h + 60 h = 71 h

Species: *Chrysomya rufifacies* (Macquart) (Diptera: Calliphoridae)

Reference: [2,4] (The data of two references are same, only [2] was cited for reducing our self-citations)

Indicator: Maximum larval body length 12.79 mm

Temperature: 28.9°C

Minimum PMI: not less than 107 h

Based on isomorphen diagrams by Hu et al. 2019 [4] and Wang 2019 [2], the egg stage is about 12 h. Based on isomegalen diagram Hu et al. 2019 [4] and Wang 2019 [2], duration of development to length 12.79 mm is about 95 h.

Total: 12 h + 95 h = 107 h

Species: *Hydrotaea spinigera* (Stein) (Diptera: Muscidae)

Reference: [2,5] (The data of two references are same, only [2] was cited for reducing our self-citations)

Indicator: Maximum larval body length 12.07 mm

Temperature: 28.9°C

Minimum PMI: not less than 90 h

Based on isomorphen diagrams by Wang 2019 [2] and Wang et al. 2020 [5], the egg stage is about 18 h. Based on isomegalen diagram by Wang 2019 [2] and Wang et al. 2020 [5], duration of development to length 12.07 mm is about 72 h.

Total: 18 h + 72 h = 90 h

Species: *Megaselia spiracularis* Schmitz (Diptera: Phoridae)

Reference: [6]

Indicator: Empty Puparia
Temperature: 28.9°C
Minimum PMI: about 273 h
Based on isomorphen diagrams by Wang et al. 2020 [6], the duration oviposition to eclosion is about 273 h. The empty puparia of *M. spiracularis* were found at scene. This means that *M. spiracularis* had completed at least one generation of development, i.e., from oviposition to eclosion. We did not estimate the age of empty puparium and we just estimated the time from oviposition to eclosion. The actual time may be longer than that.

Species: *Hermetia illucens* (L.) (Diptera: Stratyiomidae)
Reference: [2,7] (The data of two references are same, only [2] was cited for reducing our self-citations)
Indicator: Pre-pupae
Temperature: 29.1°C
Minimum PMI: about 28.80 d
Based on the table by Li et al. 2016 [7] and Wang 2019 [2], at 28 °C (the temperature nearest to that detected on site, 29.1 °C) the estimated duration from oviposition to pre-pupae: 2.8 d + 14 d + 12 d = 28.80 d

References:

1. Zhang, Y.; Wang, Y.; Yang, L.; Tao, L.; Wang, J. Development of *Chrysomya megacephala* at constant temperatures within its colony range in Yangtze River Delta region of China. *Forensic Sci. Res.* **2018**, *3*, 74–82.
2. Wang, J. *Practical Forensic Entomology*, Xi'an Jiaotong University Press: Xi'an, China, 2019.
3. Wang, Y.; Yang, L.; Zhang, Y.; Tao, L.; Wang, J. Development of *Musca domestica* at constant temperatures and the first case report of its application for estimating the minimum postmortem interval. *Forensic Sci. Int.* **2018**, *285*, 172–180.
4. Hu, G.; Wang, Y.; Sun, Y.; Zhang, Y.; Wang, M.; Wang, J. Development of *Chrysomya rufifacies* (Diptera: Calliphoridae) at Constant Temperatures Within its Colony Range in Yangtze River Delta Region of China. *J. Med. Entomol.* **2019**, *56*, 1215–1224.
5. Wang, Y.; Zhang, Y.; Wang, M.; Hu, G.; Fu, Y.; Zhi, R.; Wang, J. Development of *Hydrotaea spinigera* (Diptera: Muscidae) at Constant Temperatures and Its Significance for Estimating Postmortem Interval. *J. Med. Entomol.* **2020**, *58*, 1–8.
6. Wang, Y.; Zhang, Y.; Hu, G.; Wang, M.; Zhu, R.; Zhai, Y.; Sun, J.; Li, X.; Wang, L.; Wu, M.; Wang, J. Development of *Megaselia spiracularis* (Diptera: Phoridae) at different constant temperatures. *J. Therm. Biol.* **2020**, *93*, 102722.
7. Li, L.; Wang, Y.; Wang, J. Intra-puparial development and age estimation of forensically important *Hermetia illucens* (L.). *J. Asia-Pac. Entomol.* **2016**, *19*, 233–237.
